# DNA barcoding of sand flies (Diptera, Psychodidae, Phlebotominae) from the western Brazilian Amazon

**DOI:** 10.1371/journal.pone.0281289

**Published:** 2023-02-02

**Authors:** Israel de Souza Pinto, Bruno Leite Rodrigues, Thais de Araujo-Pereira, Paloma Helena Fernandes Shimabukuro, Daniela de Pita-Pereira, Constança Britto, Reginaldo Peçanha Brazil

**Affiliations:** 1 Departamento de Ciências Biológicas, Instituto Federal de Educação, Ciência e Tecnologia do Pará, Itaituba, Pará, Brasil; 2 Programa de Pós-Graduação em Saúde Pública, Faculdade de Saúde Pública, Universidade de São Paulo, São Paulo, São Paulo, Brasil; 3 Laboratório de Biologia Molecular e Doenças Endêmicas, Instituto Oswaldo Cruz, Fundação Oswaldo Cruz, Rio de Janeiro, Rio de Janeiro, Brasil; 4 Coleção de Flebotomíneos, Grupo de Estudos em Leishmanioses, Instituto René Rachou, Fundação Oswaldo Cruz, Belo Horizonte, Minas Gerais, Brasil; 5 Laboratório de Doenças Parasitárias, Instituto Oswaldo Cruz, Fundação Oswaldo Cruz, Rio de Janeiro, Rio de Janeiro, Brasil; Federal University of Mato Grosso do Sul, BRAZIL

## Abstract

The subfamily Phlebotominae comprises important insects for public health. The use of complementary tools such as molecular taxonomy is necessary for interspecific delimitation and/or discovery of cryptic species. Here, we evaluated the DNA barcoding tool to identify different species in the southwestern Brazilian Amazon. For this, we collected sand flies in forest fragments along the highway BR-317, in the municipality of Brasiléia, state of Acre, Brazil. The specimens were DNA-barcoded using a fragment of the *cytochrome c oxidase subunit I* (*COI*) gene. The sequences were analyzed to generate K2P pairwise genetic distances and a Neighbour-joining tree. The sand fly barcodes were also clustered into Molecular Operation Taxonomic Units (MOTU) using Automatic Barcode Gap Discovery (ABGD) approach. A total of 59 *COI* sequences comprising 22 nominal species and ten genera were generated. Of these, 11 species had not been sequenced before, thus being new *COI* sequences to science. Intraspecific genetic distances ranged between 0 and 4.9%, with *Pintomyia serrana* presenting the highest values of genetic distance, in addition to having been partitioned into three MOTUs. Regarding the distances to the nearest neighbour, all species present higher values in relation to the maximum intraspecific distance, in addition to forming well supported clusters in the neighbour-joining analysis. The DNA barcoding approach is useful for the molecular identification of sand flies from Brasiléia, state of Acre, and was efficient in detecting cryptic diversity of five species which can be confirmed in future studies using an integrative approach. We also generated new *COI* barcodes for *Trichophoromyia auraensis*, *Nyssomyia shawi*, and *Psychodopygus paraensis*, which may play a role in the transmission of *Leishmania* spp. in the Brazilian Amazon.

## Introduction

Sand flies (Diptera, Psychodidae, Phlebotominae) are the main insects involved in the transmission of *Leishmania* spp. parasites to humans and other animals [1, 2], comprising about 500 species and 23 genera in Neotropical regions [3, 4]. The subfamily Phlebotominae is widely distributed in Brazil, and the Amazon biome presents the largest number of species recorded, including most of the genera *Bichromomyia*, *Nyssomyia*, *Psychodopygus*, and *Trichophoromyia* which includes incriminated vectors of the etiological agents of cutaneous leishmaniasis (CL) [1, 5]. The state of Acre, located in the southwest of the Brazilian Amazon, has a great diversity of sand fly species associated to different habitats, reservoirs, and protozoans [6–8], totaling 116 species of these insects [9], which increases the relevance of correct entomological monitoring in these localities.

Despite the relevance to public health, the species-level identification of sand flies can be hampered due to the complexity of morphological characters, and the lack of differences between close-related taxa [4]. There are some complexes of cryptic species reported among sand flies [10], and taxa that are only known based on the morphological information from a single sex [4]. For this reason, integrative taxonomy approaches may use complementary tools to elucidate interspecific boundaries [11, 12]. One of these tools–the DNA barcoding–aims to assist in the species-level identification through the sequencing of a fragment of the *cytochrome c oxidase subunit I* (*COI*) gene, which allows the identification of closely related species [13]. Indeed, the sand fly systematics and taxonomy using DNA sequences are relatively understudied, and only about a third of the nominal species has been processed for any molecular marker, and a quarter of them for the *COI* gene [14]. In Brazil, some efforts have been made to evaluate DNA barcodes as effective in delimiting several species of different genera [15, 16], but information on Amazonian taxa is lacking, where some studies focused on a few numbers of close-related species [17–19].

Therefore, we aim to evaluate and make available new *COI*-barcodes for sand flies from a highly diverse region in the Brazilian Amazon, which may be of great relevance for future monitoring of these insects. Sand flies were collected in the municipality of Brasiléia, in the south of Acre state, in areas of current transmission of *Leishmania* spp. causing cutaneous leishmaniasis according to human cases registered in the recent years by the National Health Foundation (FUNASA) and the local Fernando Azevedo Correia Center of Health of Brasiléia [7]. In this study, we generated *COI* sequences for sand fly species already processed from other locations, which may help to elucidate the phylogeographic patterns and species discovery within these taxa in upcoming studies.

## Materials and methods

### Study area and collection

**Fig 1:**
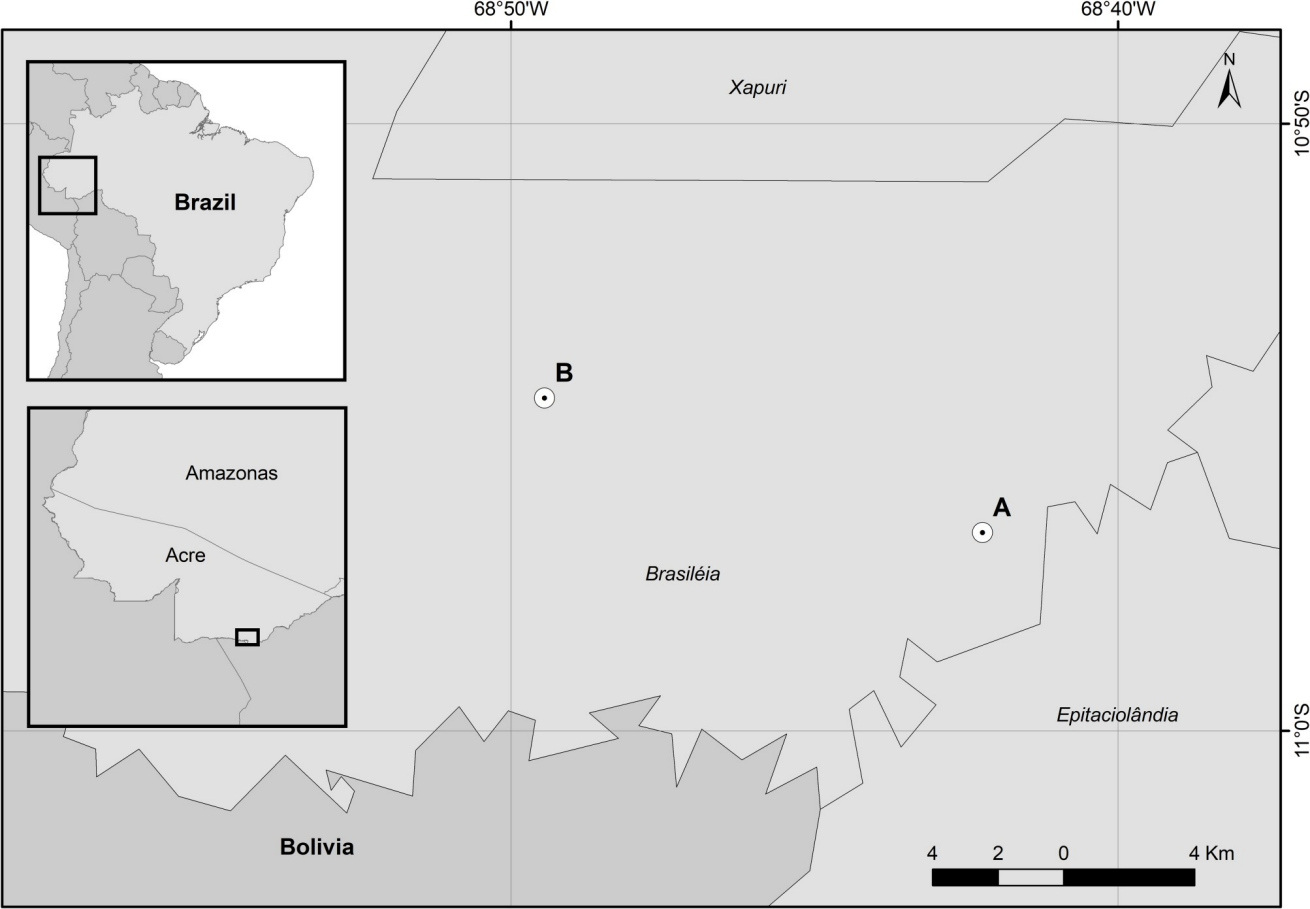
Map of the study area. Map of Brazil highlighting the state of Acre and the municipality of Brasiléia. The collection sites A and B correspond to *Ramal* 04 (km 04) and *Ramal* 13 (km 13) of the BR-317 Trans Pacific highway to Brasiléia, respectively.

For the present study, sand flies were obtained from two collection sites situated in the rural zone of the municipality of Brasiléia, in areas transversely positioned along the BR-317 Trans Pacific highway connecting Brasiléia and the municipality of Assis Brasil, in the state of Acre, Brazil. The collection sites were identified as *Ramal* (or branch) based on the distance in kilometers of BR-317 Trans Pacific highway from the center of Brasiléia (Fig 1), and were selected due to the high incidence of human cutaneous leishmaniasis cases in forested areas close to residences in rural areas [7]. Collections were carried out in private areas after receiving permission from the landowners. Sand fly collections were performed under a permanent license of the Brazilian *Sistema de Autorização e Informação em Biodiversidade* (SISBIO) for the capture of zoological material (permit 32669–4).

The municipality of Brasiléia, which has an estimated population of 27,000 inhabitants [20], is located in the south of Acre on the border with the municipality of Cobija–Bolivia and the Brazilian municipalities of Epitaciolândia, Assis Brasil, Sena Madureira and Xapuri. The insects were collected using HP light traps that operated overnight inside high-density forested areas, with the following coordinates: A—(S 10°56’44”; W 68°42’14”), and B—(S 10°54’31”; W 68°49’27”). A small container (220 mL) with 80% ethanol was connected to each trap to store the insects while they were being captured [21]. Male sand flies were screened and had their legs dissected for later DNA extraction, while the rest of the body was mounted between slides and coverslips for morphological identification according to the key and classification of [4]. The abbreviations of generic names follow the proposed by [22].

### DNA barcoding

To generate the DNA barcodes, total DNA was extracted from the legs of each male sand fly specimen. All the legs available for each individual were placed in tubes (one for each specimen), and DNA extraction was performed. Genomic DNA was used to amplify/sequencing the DNA barcoding region of the *cytochrome c oxidase subunit I* (*COI*) gene, according to [15]. In summary, a rapid DNA extraction method for DNA amplification was performed as reported by [23]. To amplify a 658 bp fragment of the *COI* gene, PCR was carried out in a final volume of 50 μL reaction mixture containing 2 μL of genomic DNA template, 25 μL 2X Promega Go Taq Green™ Master Mix (Promega, Madison, WI, USA), and the primers LCO1490 (5’-GGTCAACAAATCATAAAGATATTGG-3’) and HCO2198 (5’-TAAACTTCAGGGTGACCAAAAAATCA-3’) in final concentrations of 1.0 μM each [24]. The cycling conditions consisted of an initial denaturation step of 95°C for 3 min, followed by 37 cycles of 95°C for 1 min, 57°C for 1 min, 72°C for 1.5 min, and final extension at 72°C for 7 min. The amplified fragments were separated by agarose (2%) gel electrophoresis and purified using Illustra GFX PCR DNA™ and Gel Band Purification Kit (GE Healthcare, Pittsburgh, PA, USA). The purified fragments were sequenced bidirectionally using the same PCR primers for the *COI* gene. Reactions were performed using Big Dye Terminator v.3.1 Cycle Sequencing (Applied Biosystems Foster City, USA) following the manufacturer’s specifications and assayed in the Sanger ABI 3730 DNA analyzer at Fundação Oswaldo Cruz (PDTIS/Fiocruz), Rio de Janeiro, Brazil. The generated sequences were submitted to GenBank nucleotide sequence database [25], and have been assigned the Accession Numbers OP346789-OP346847. In addition, our dataset was also submitted to the Barcode of Life Data System (BOLD) [26] for further analysis.

Sequences were initially visualized and edited in the software BioEdit v5.0.9.0 [27, 28], and then the alignment was performed using ClustalW [29] as implemented in the Mega v7 [30]. Visual inspection of the alignment indicated the absence of pseudogenes and/or nuclear copies of mitochondrial origin (NUMTs). The maximum intraspecific pairwise genetic distances, in addition of the minimum distance to the nearest-neighbour of each nominal species (interspecific), were generated using Kimura 2-parameters model (K2P) in the BOLD Systems environment.

In order to check the clustering pattern and the utility of *COI*-barcodes for the molecular identification of sand flies from the southwestern Brazilian Amazon, we combined our new sequences to those previously processed in GenBank for the sampled nominal species. Then, we reconstructed a neighbour-joining tree (NJ) for the combined dataset in the software MEGA v7 with 1,000 bootstraps pseudoreplicates and K2P model. The sequences of each specimen were also identified at the Molecular Operational Taxonomic Unit (MOTU) level [31], using the Automatic Barcode Gap Discovery (ABGD) algorithm [32] which is available at https://bioinfo.mnhn.fr/abi/public/abgd/abgdweb.html (last access at 13^th^ Jun 2022). For this, we used the combined dataset with no missing data, and the parameters: Pmin = 0.001, Pmax = 0.1, X = 1.0, and K2P model. After the run, we considered the recursive partitions with prior maximal distances between 1% and 2.5%.

### Ethics statement

Access to genetic heritage has been registered into the *Sistema Nacional de Gestão do Patrimônio Genético e do Conhecimento Tradicional Associado*—SisGen (A41DBDD). Sand fly collection was carried out under a permanent license provided by the *Sistema de Autorização e Informação em Biodiversidade*—SISBIO (permit 32669–4).

## Results

In total, we generated 59 *COI* sequences for the sand flies sampling from the state of Acre (n = 59), southwestern of the Brazilian Amazon. These sequences are distributed in 22 species and ten genera (Table 1), and some of these *COI* sequences, representing 11 species, are new to science: *Evandromyia saulensis* (Floch & Abonnenc, 1944), *Lutzomyia flabellata* Martins & Silva, 1964, *Nyssomyia shawi* (Fraiha, Ward & Ready, 1981), *Psathyromyia dendrophyla* (Mangabeira, 1942), *Psychodopygus amazonensis* (Root, 1934), *Psychodopygus claustrei* (Abonnenc, Léger & Fauran, 1979), *Psychodopygus francoisleponti* Zapata, Depaquit & León 2012, *Psychodopygus paraensis* (Costa Lima, 1941), *Trichophoromyia auraensis* (Mangabeira, 1942), *Trichophoromyia octavioi* (Vargas, 1949), and *Trichopygomyia dasypodogeton* (Castro, 1939). In addition, we also sequenced previously published species for *COI* gene, but which are new to the state of Acre: *Evandromyia bacula* (Martins, Falcão & Silva, 1965), *Nyssomyia antunesi* (Coutinho, 1939), *Nyssomyia whitmani* (Antunes & Coutinho, 1939), *Pintomyia serrana* (Damasceno & Arouck, 1949), *Pressatia choti* (Floch & Abonnenc, 1941), *Psathyromyia abonnenci* (Floch & Chassignet, 1947), *Psathyromyia aragaoi* (Costa Lima, 1932), *Psychodopygus carrerai carrerai* (Barretto, 1946), *Psychodopygus davisi* (Root 1934), *Psychodopygus hirsutus* (Mangabeira, 1942), and *Viannamyia furcata* (Mangabeira, 1941).

**Table 1:** List of nominal sand fly species collected in the western Brazilian Amazon which generate DNA barcode sequences. Number of sequences, intraspecific and interspecific (to the nearest neighbour) K2P genetic distances are presented.

Species	Number of sequences	Mean Intraspecific distance	Max Intraspecific distance	Distance to Nearest Neighbour
*Evandromyia bacula*	1	N/A	N/A	15.30
*Evandromyia saulensis* *	2	2.17	2.17	16.62
*Lutzomyia flabellata* *	1	N/A	N/A	11.83
*Nyssomyia antunesi*	2	0.46	0.46	5.23
*Nyssomyia shawi* *	1	N/A	N/A	11.48
*Nyssomyia whitmani*	3	0.51	0.61	5.23
*Pintomyia serrana*	6	3.12	4.9	11.13
*Pressatia choti*	3	0.2	0.3	14.39
*Psathyromyia abonnenci*	1	N/A	N/A	10.79
*Psathyromyia aragaoi*	1	N/A	N/A	12.19
*Psathyromyia dendrophyla* *	2	0.61	0.61	10.79
*Psychodopygus amazonensis* *	1	N/A	N/A	8.56
*Psychodopygus carrerai*	3	1.6	2.32	9.39
*Psychodopygus claustrei* *	2	0	0	7.24
*Psychodopygus davisi*	7	1.09	2.33	7.24
*Psychodopygus francoisleponti* *	3	0.1	0.15	11.32
*Psychodopygus hirsutus*	4	2.1	3.29	9.57
*Psychodopygus paraensis* *	4	0.54	1.07	9.39
*Trichophoromyia auraensis* *	5	0	0	2.49
*Trichophoromyia octavioi* *	3	0.61	0.77	2.49
*Trichopygomyia dasypodogeton* *	1	N/A	N/A	11.85
*Viannamyia furcata*	3	1.86	2.8	10.78

N/A, not applicable.

* species that were *COI*-DNA barcoded for the first time.

Our new *COI*-barcode sequences have its full-length of 658 bp. For this dataset, the intraspecific mean and maximum K2P distances ranged from 0.0% to 3.1%, and 0.0% to 4.9%, respectively (Table 1). Most species did not obtain intraspecific distances greater than 3%, with the exception of *Pi*. *serrana* (maximum K2P = 4.9%). On the other hand, the minimum K2P distances to the nearest neighbour (interspecific) ranged from 2.49% to 16.62%, with a notable discrepancy in the species of the *Trichophoromyia* genus, which achieved interspecific values lower than the intraspecific comparisons of some species. Despite that, all species present higher values in relation to the maximum intraspecific distance (Table 1).

**Fig 2:**
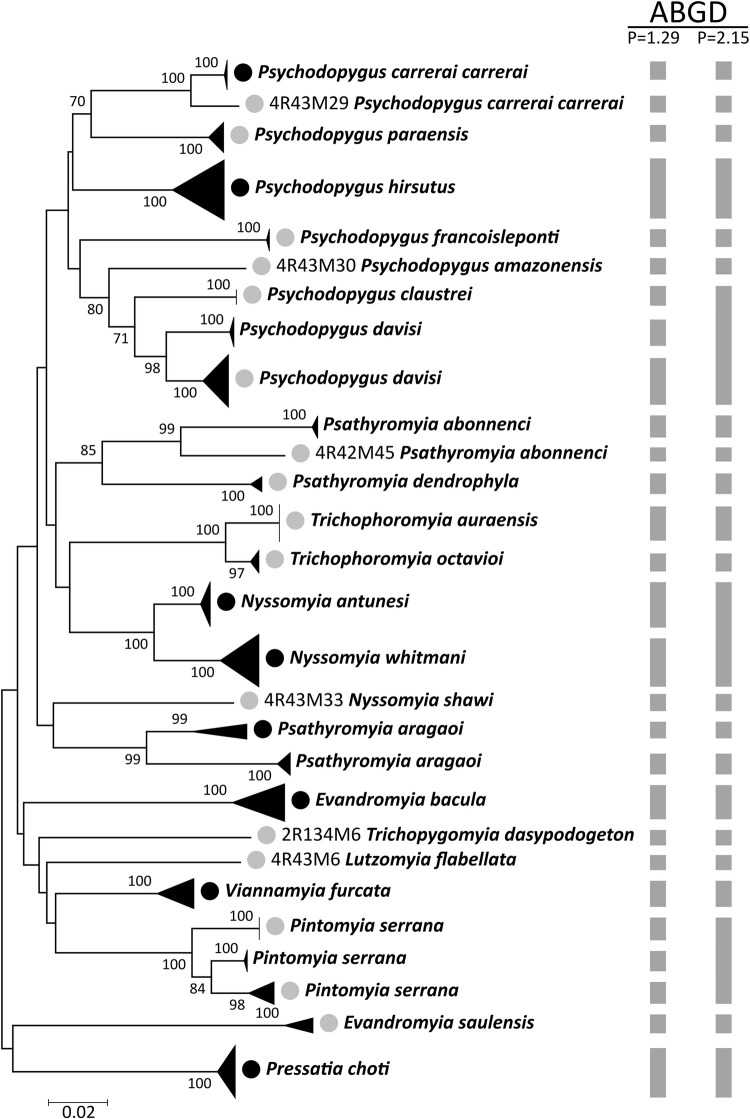
Neighbour-joining gene tree of sand fly *COI* barcode sequences.

The combined dataset comprised our 59 sequences plus 37 conspecific barcodes previously deposited and available in GenBank database, totaling 96 sequences. The neighbour-joining phenogram clustered the sequences according to the sampled nominal species with high values of bootstrap support (i.e., >90) (Fig 2 and S1 Fig). This pattern continues to be seen regarding those species that have conspecific sequences from GenBank. Further, the species *Ps*. *carrerai carrerai*, *Ps*. *davisi*, *Pa*. *abonnenci*, *Pa*. *aragaoi*, and *Pi*. *serrana* splitted in at least two different and well-supported clusters, but none of the nominal species were merged with other ones (Fig 2 and S1 Fig).

The analyzed species comprise specimens from the state of Acre and their conspecific sequences extracted from GenBank (when available). The number near nodes indicates bootstrap above 70. Tip labels marked with grey circles indicate clades with sequences generated in this study (state of Acre, Brazil), while black circles indicate those with Acre plus GenBank sequences. The unmarked clades have GenBank sequences only. The grey bar represents the species delimitation made by the ABGD algorithm.

The species delimitation made by ABGD algorithm, sorted the sequences into 28 and 23 MOTUs in the recursive partitions of P = 1.29 and P = 2.15, respectively (Fig 2 and S1 Fig). The most conservative scenario (P = 2.15) merged the species pairs *Ps*. *davisi*/*Ps*. *claustrei* and *Ny*. *antunesi*/*Ny*. *whitmani* into the same MOTU each, while the other nominal species formed at least one MOTU for each one (Fig 2 and S1 Fig). In contrast, the ABGD partition with P = 1.29 correctly delimited the above-mentioned species pairs, in addition to splitting specimens of *Ps*. *carrerai carrerai*, *Ps*. *davisi*, *Pa*. *abonnenci*, *Pa*. *aragaoi* into two different MOTUs, and *Pi*. *serrana* into three MOTUs (Fig 2 and S1 Fig).

## Discussion

Here we provide the first *COI* DNA barcodes of sand flies from the state of Acre, located in the western Brazilian Amazon. These records comprise some sequences of species that were never analyzed for this marker, and can be used for future studies regarding the molecular identification and phylogeographic studies of this group. Further, we demonstrated that *COI*-barcodes have enough information for the species delimitation of several species from western Brazilian Amazon, and are useful in detecting cryptic diversity within these taxa, which may represent different cryptic species or structured populations. The relevance of these findings for entomological monitoring of sand flies, and for public health of areas with the transmission of leishmaniasis should be accessed in future studies.

The results of genetic divergences and the clustering pattern of neighbour-joining tree indicate that all species analyzed in this study can be correctly identified by DNA barcodes. Despite this, the species delimitation made by ABGD algorithm merged some close-related species, such as *Ps*. *davisi*/*Ps*. *claustrei* and *Ny*. *antunesi*/*Ny*. *whitmani*, when using more conservative parameters. Similarly, *Ny*. *whitmani* and *Ny*. *intermedia* from southeast Brazil are not correctly delimited depending on the parameters used in the ABGD analysis [15], and other species of this genus are merged into the same MOTU while analyzing with more diverged species [16]. These two genera, especially *Nyssomyia*, have little *COI* genetic differentiation between the nominal species compared to other sand fly genera, and may have different species merged into the same MOTU using distance-based methods such as ABGD. These results indicate that the best ABGD partition scheme for our dataset is the one with P = 1.29, which correctly delimited all the analyzed species, sometimes partitioning the same taxon into different MOTUs.

Studies that aim to provide sand fly DNA barcodes in the Brazilian Amazon are scarce, despite the great diversity of species found in the region. In most cases, the sequencing of *COI* fragments is performed for a few species from different locations, mainly because they are related to the transmission of pathogens, such as the species *Nyssomyia umbratilis* [17, 18] and *Bichromomyia* spp. [19]. Our study represented the first effort to sample different sand fly species and genera in the Brazilian Amazon, accounting for 22 analyzed species. Despite this, the state of Acre has 116 recorded nominal species of these insects [9], and our dataset comprises only about 19% of this diversity in a specific locality, demonstrating that future studies should assess the real usefulness of *COI* barcodes in the identification of different sand flies in Acre and Brazil, both in terms of different species and locations.

We generated for the first time the *COI* barcode sequences for 11 sand fly species, including some that are of relevance for the transmission of *Leishmania* pathogens in South America. Of these, the sequencing of *Th*. *auraensis* and *Th*. *octavioi* are especially important because the first is considered a putative vector of *Leishmania* (*Viannia*) in the Amazon region, including the state of Acre [33–35], in addition of their females being isomorphic [4], so the male-female association using barcodes may be implemented to correctly identify these insects in upcoming studies (e.g., [15, 36, 37]). We are providing the first *COI* barcodes for two vector species–*Ny*. *shawi* and *Ps*. *paraensis*; the former being associated to the transmission of *Leishmania guyanensis* in Bolivia [1], and the latter is considered the vector of *Leishmania naiffi* in Brazil [1, 38]. These and the other new barcode records are important for understanding the taxonomy and systematics of sand flies in the Americas, and could be used for future molecular identification of these taxa.

We revealed cryptic diversity in five sand fly species collected in the state of Acre: *Ps*. *carrerai carrerai*, *Ps*. *davisi*, *Pa*. *abonnenci*, *Pa*. *aragaoi*, and *Pi*. *serrana*. In the cases of *Ps*. *davisi*, *Pa*. *abonnenci*, and *Pa*. *aragaoi*, the barcode sequences were splitted into two MOTUs, one of them containing individuals from the state of Acre and the other with sequences from southeastern Brazil (*Ps*. *davisi*; [15]) or Colombia (*Pa*. *abonnenci* and *Pa*. *aragaoi*; [39, 40]). This clustering pattern seems to be related to the geographic distances of the analyzed specimens in addition of the landscape heterogeneity within Brazil and South America, which may have promoted the isolation of these populations and interruption of the gene flow between them [41], a result previously seen when analyzing sand fly barcode sequences from broader spatial scales in Brazil [16]. On the other hand, *Ps*. *carrerai carrerai* presented an allopatric cryptic diversity, with sequences being splitted within the same locality (Brasiléia, state of Acre), while *Pi*. *serrana* splitted into three MOTUs and presents both allopatric and sympatric patterns of cryptic diversity. Whether or not these different MOTUs within nominal species are in fact different taxa, should be evaluated using integrative taxonomy approaches.

In summary, the DNA barcoding strategy is useful for the molecular identification of sand flies from western Brazilian Amazon, and was efficient in detecting cryptic diversity of *Pi*. *serrana*, which can be confirmed in future studies using an integrative approach. Further, it was generated remarkable *COI*-barcode records, like *Th*. *auraensis*, *Ny*. *shawi*, and *Ps*. *paraensis* which play a role in the transmission of *Leishmania* spp. in the Brazilian Amazon.

## Supporting information

S1 Fig Expanded neighbour-joining gene tree of sand fly *COI* barcode sequences. The analyzed species comprise specimens from the state of Acre and their conspecific sequences extracted from GenBank (when available). The number near nodes indicates bootstrap values greater than 70. Tip labels marked with grey circles indicate clades with sequences generated in this study (state of Acre, Brazil). The unmarked clades have GenBank sequences only. The grey bar represents the species delimitation made by the ABGD algorithm.
(TIF)
