# Supplementary figures and images for: DNA barcoding of sand flies (Diptera, Psychodidae, Phlebotominae) from the western Brazilian Amazon
Source: PLoS One. 2023 Feb 2;18(2):e0281289. doi: 10.1371/journal.pone.0281289 (PMC9894394; doi:10.1371/journal.pone.0281289)

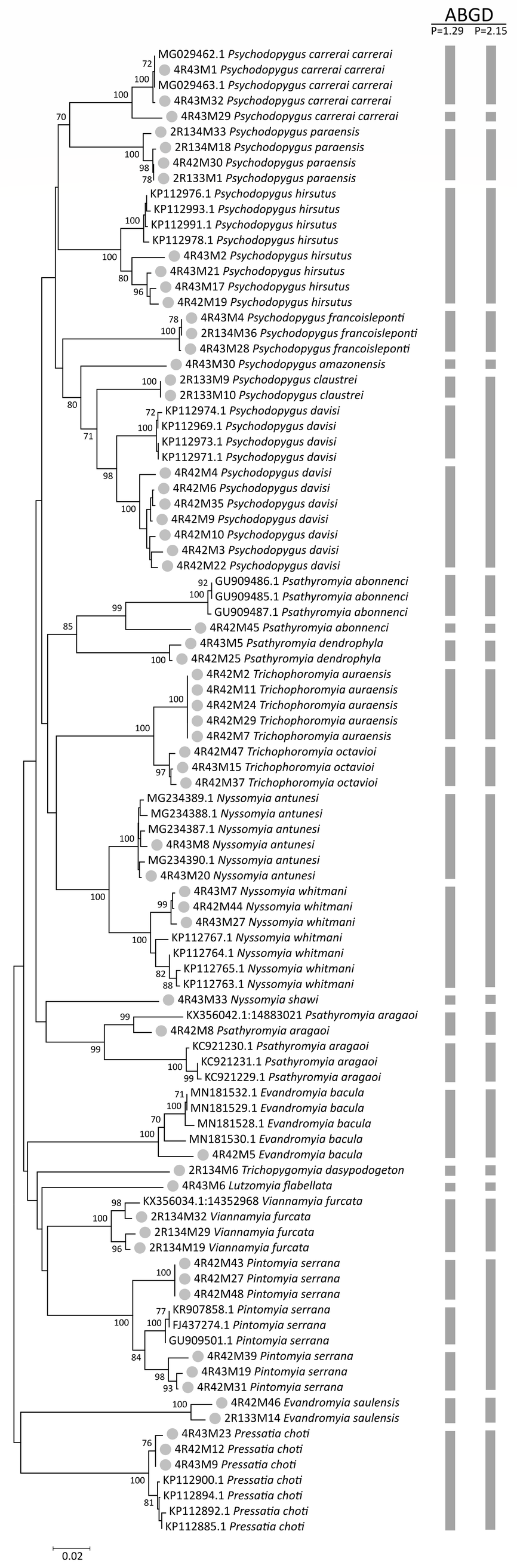

Supplement: S1 Fig — The analyzed species comprise specimens from the state of Acre and their conspecific sequences extracted from GenBank (when available). The number near nodes indicates bootstrap values greater than 70. Tip labels marked with grey circles indicate clades with sequences generated in this study (state of Acre, Brazil). The unmarked clades have GenBank sequences only. The grey bar represents the species delimitation made by the ABGD algorithm. (TIF) [file pone.0281289.s001.tif]
